# Total posterior quadrant disconnection for drug‐resistant epilepsy in children

**DOI:** 10.1002/epi4.13044

**Published:** 2024-09-19

**Authors:** Hao Yu, Chang Liu, Yu Sun, Yao Wang, Qingzhu Liu, Taoyun Ji, Shuang Wang, Xiaoyan Liu, Yuwu Jiang, Ye Wu, Lixin Cai

**Affiliations:** ^1^ Pediatric Epilepsy Center Peking University First Hospital Beijing China

**Keywords:** children, development, drug‐resistant epilepsy, posterior quadrant disconnection

## Abstract

**Objective:**

To assess seizure outcomes, prognostic factors, and developmental changes in children undergoing total posterior quadrant disconnection (PQD) for drug‐resistant epilepsy (DRE).

**Methods:**

We conducted a retrospective analysis of the clinical data of children with DRE who underwent total PQD surgery. The study focused on Engel's classification for seizure outcomes, exploring correlation of preoperative data and surgical effectiveness, and predictors of seizure prognosis. It involved a comparative analysis of developmental levels pre‐ and 3 months postoperatively using Griffiths Mental Development Scales‐China (GMDS‐C), and the correlation between clinical characteristics and GMDS‐C results.

**Results:**

Out of 61 pediatric patients, 70.5% showed no seizure recurrence postoperatively. In the univariate analysis, interictal electroencephalogram (EEG), magnetic resonance imaging (MRI), fluorodeoxyglucose positron emission tomography (FDG‐PET), and acute postoperative seizure (APOS) were significantly related to surgical prognosis. In multivariate analysis, interictal EEG and APOS were identified as predictors of seizure prognosis. Survival analysis indicated significant associations between MRI, interictal EEG, FDG‐PET, APOS and postoperative seizure occurrence. Preoperative GMDS‐C levels were significantly correlated with epilepsy duration, seizure frequency, interictal EEG, and FDG‐PET. GMDS‐C scores improved postoperatively, while developmental quotients remained stable.

**Significance:**

For patients with structural abnormalities in the entire posterior quadrant, thorough preoperative assessment and timely total PQD surgery can effectively control seizures without causing neurological development deterioration. APOS and interictal EEG abnormalities beyond the posterior quadrant are predictors for seizure prognosis but should not be deemed contraindications for surgery.

**Plain Language Summary:**

Due to lack of analysis on pediatric total PQD cases, 61 pediatric patients who underwent total PQD surgery were retrospectively enrolled. Seizure and development results were collected and analyzed as dependent variables. The study found that 70.5% of patients were seizure‐free and showed development improvement, with no deaths or severe complications reported. Prognosis predictors included APOS and interictal EEG abnormalities beyond the posterior quadrant.

**Key points**

- 70.5% (43/61) of patients showed no seizure recurrence after total PQD surgery.
- For patients with structural abnormalities in the entire posterior quadrant, thorough preoperative assessment and timely total PQD surgery can effectively stop seizures without causing deterioration in neurological development.
- APOS and interictal EEG abnormalities beyond the posterior quadrant are predictive factors for seizure prognosis and should not be considered contraindications for surgery.

## 1 INTRODUCTION

The proportion of children undergoing multilobar epilepsy surgery is higher than that of adults.^1^, ^2^, ^3^, ^4^ Posterior quadrant disconnection (PQD) is a common and effective surgical procedure for treating drug‐resistant epilepsy (DRE). The most common etiology is malformation of cortical development (MCD) in the posterior regions of the brain, with posterior quadrant MCD affecting 3%–15% of individuals with multilobar MCD.^5^

In previous literature, numerous studies have reported overwhelmingly favorable outcomes for the surgical treatment of PQD in epilepsy. However, there has been a paucity of research specifically focusing on pediatric cases.^5^, ^6^, ^7^, ^8^, ^9^ A review article published 2 years ago summarized nine studies on PQD surgery, revealing an overall postoperative seizure‐free rate of 66.4%. The review concluded that total PQD demonstrated superior efficacy compared to partial PQD.^10^ Several studies have combined and analyzed both surgical approaches; however, the procedures for partial PQD vary significantly due to lesion size and location, resulting in heterogeneity in statistical analysis. To date, there are no retrospective studies investigating a substantial number of pediatric patients treated with total PQD for drug‐resistant epilepsy (DRE).

## 2 MATERIALS AND METHODS

### 2.1 Inclusion criteria

Between July 2015 and December 2019, a total of 61 pediatric patients with DRE who were treated with total posterior quadrant disconnection (PQD) (disconnection of the whole temporal, parietal and occipital lobes excluding the posterior central gyrus, but in some circumstances, partial operculum or posterior insular resection were included) at the Pediatric Epilepsy Center of Peking University First Hospital (PKFHPEC) were enrolled. These patients were selected from 110 individuals with posterior quadrant epileptogenic lesions. The inclusion criteria for this study were: (a) patients diagnosed with DRE based on the criteria defined by the International League Against Epilepsy (ILAE)^11^ or those with frequent seizures despite a shorter epilepsy duration but with definite structural epileptogenic lesions located in the posterior quadrant on MRI; (b) patients younger than 18 years old who underwent multidisciplinary preoperative evaluation and total PQD surgery; and (c) patients with a postoperative follow‐up period exceeding 3 years.

### 2.2 Pre‐surgery examinations

All patients underwent preoperative assessment, which included long‐term scalp EEG using the 10–20 system, three‐dimensional high‐resolution MR (3.0 T), and fluorodeoxyglucose positron emission tomography (FDG‐PET) brain scans. MRI‐PET coregistration and fusion were performed to further delineate the extent of resection or disconnection. In cases where defining the boundaries of the lesion proved challenging, invasive intracranial EEG evaluation was considered. The findings from EEG, MRI, and FDG‐PET were interpreted by relevant professionals other than the analyst to ensure objectivity.

### 2.3 Surgical procedure

The primary surgical steps included the following: first, a partial resection of the parietal lobule was performed to expose the posterior insula, posterior insular point, and posterior part of the inferior circular sulcus. This step facilitated the subsequent disconnection, and the excised brain tissue was sent for pathological examination. The dissection continued along the posterior part of the central sulcus toward the lateral ventricle, followed by disconnection of the posterior portion of the corpus callosum and fornix. Finally, the medial structures of the temporal lobe were excised. This surgical approach was adapted from Daniel's technique.^12^ For patients with lesions involving the central posterior sulcus or gyrus, motor evoked potential (MEP) and somatosensory evoked potential (SEP) assessments were performed during surgery to ensure safety and efficacy. All procedures were carried out by the same experienced neurosurgeon and their team members.

### 2.4 Seizure outcome

The Engel classification was utilized for the evaluation of postoperative seizure outcomes.^13^ Seizures occurring within the first week after surgery were categorized as acute postoperative seizures (APOS).^14^ In the Kaplan–Meier survival analysis, the time of the first seizure occurring after the first week post‐surgery was considered the time of seizure recurrence. The last antiseizure medication (ASM) was typically discontinued at least 2 years postoperatively, with routine EEG examinations conducted at 3 months, 1 year, and annually thereafter.

### 2.5 Developmental assessment

Developmental assessment was conducted using the Chinese Griffiths Mental Development Scales (GMDS‐C). GMDS‐C assessments were performed both before and 3 months after surgery. A comparison was made in terms of the average raw scores and developmental quotients (DQ) in five different domains of the GMDS‐C before and after surgery. Additionally, the correlation between clinical characteristics and preoperative GMDS‐C scores was analyzed to identify factors influencing developmental outcomes.

### 2.6 Ethics and informed consent

This study was approved by the Ethics Committee of Peking University First Hospital. Written consent was obtained from the parents of each subject to utilize their children's data for research purposes.

### 2.7 Statistical analysis

The dependent variables included seizure outcomes and developmental changes. The independent variables included clinical characteristics, such as MRI, EEG, FDG‐PET, APOS, etc. Univariate and multivariate analysis were conducted consecutively.

The Shapiro–Wilk test was used as the formal test for normality. The Wilcoxon rank‐sum test was employed for univariate analysis of skewed distribution continuous variables, while Pearson's chi‐squared test or Fisher's exact test was used for categorical variables. Variables with a significance level <0.05 in the initial univariate analysis were subsequently tested in multivariate logistic regression analysis. A Kaplan–Meier survival curve was utilized to estimate the probability of seizure freedom over time, and statistical significance was tested using the log‐rank test. For the correlation between presurgical GMDS‐C and characteristics, Fisher's exact test and Spearman correlation were employed. The paired *T* test was used for comparative analysis of pre‐ and postoperative GMDS‐C. Statistical software was performed using R 4.2.2 (Lucent Technologies, New Jersey, USA).

## 3 RESULTS

This study included 61 eligible patients, comprising 36 males. The median age at the time of surgery was 2.39 years (0.77–15.29 years). The median age at the time of onset was 0.47 years (0.00–7.62 years). The median duration of the disease was 1.87 years (0.19–9.92 years).

### 3.1 Presurgical evaluation

In this study, 44.3% experienced seizures more than 10 times per day, 47.5% had fewer than 10 seizures daily and 8.2% had seizures once a week. Overall, over 90% of patients had daily seizures.

#### 3.1.1 Seizure semiology

41.0% had one type of focal nonspasm seizure, 29.5% had only spasm seizures, 18.0% had both spasms and another type of focal seizure, and 11.5% had three or more seizure types (with or without spasm seizures). Overall, 55.7% of patients (55.7%) had spasms.

#### 3.1.2 EEG

Analysis of interictal EEG results indicated that 67.2% of patients had abnormal discharges localized within the posterior quadrant region. Another 11.5% showed abnormal discharges outside of the disconnection area but in the ipsilateral hemisphere, and 21.3% had widespread or multifocal bilateral abnormal discharges. In terms of ictal EEG, 55.7% of patients had seizures originating within the disconnection area, 13.1% had seizures beginning in the ipsilateral hemisphere without a clear location, and 31.2% of cases, the side of seizure onset could not be determined.

#### 3.1.3 MRI

In all patients, MRI scans revealed clear lesions. Specifically, 77.1% had lesions confined to the surgical disconnection extension, 18.0% had lesions extending beyond the disconnection area but limited to the ipsilateral hemisphere, and 4.9% had lesions involving both hemispheres, with minor lesions on the contralateral hemisphere.

#### 3.1.4 FDG‐PET

Abnormal brain tissue metabolism was detected solely within the surgical disconnection area in 55.7% of patients, beyond the disconnection area but limited to the same hemisphere as the lesion in 36.1%, and in both hemispheres in 8.2%. Except for one patient showing hypermetabolism, the remaining 60 patients displayed hypometabolism (Table 1).

**TABLE 1:** Clinical characteristics and univariate analysis of seizure outcomes.

Characteristic	Overall, *N* = 61^a^	ENGEL I, *N* = 43^a^	ENGEL II–IV, *N* = 18^a^	*p*‐value^b^
Sex				
Male	36 (59.0%)	23 (53.5%)	13 (72.2%)	0.175
Female	25 (41.0%)	20 (46.5%)	5 (27.8%)	
Onset age (year)	0.47 (0.00–7.62)	0.49 (0.00–7.62)	0.44 (0.01–6.26)	0.7698
Surgery age (year)	2.39 (0.77–15.29)	2.59 (0.77–10.96)	2.37 (1.09–15.29)	0.6779
Duration (year)	1.87 (0.19–9.92)	1.88 (0.19–8.38)	1.60 (0.87–9.92)	0.8681
Semiology				
Focal	25 (41.0%)	21 (48.8%)	4 (22.2%)	0.120
Spasms	18 (29.5%)	11 (25.6%)	7 (38.9%)	
Spasms+focal	11 (18.0%)	8 (18.6%)	3 (16.7%)	
Multifocal	7 (11.5%)	3 (7.0%)	4 (22.2%)	
Spasms				
Present	34 (55.7%)	21 (48.8%)	13 (72.2%)	0.094
None	27 (44.3%)	22 (51.2%)	5 (27.8%)	
Frequency				
>10 per day	27 (44.3%)	18 (41.9%)	9 (50.0%)	0.915
<10 per day	29 (47.5%)	21 (48.8%)	8 (44.4%)	
Per week	5 (8.2%)	4 (9.3%)	1 (5.6%)	
Side				
L	32 (52.5%)	24 (55.8%)	8 (44.4%)	0.417
R	29 (47.5%)	19 (44.2%)	10 (55.6%)	
EEG onset lobe				
disco‐PQ	34 (55.7%)	26 (60.5%)	8 (44.4%)	0.324
ipsi‐hemi	8 (13.1%)	4 (9.3%)	4 (22.2%)	
Uncertain	19 (31.2%)	13 (30.2%)	6 (33.3%)	
Interictal EEG lobe				
disco‐PQ	41 (67.2%)	34 (79.1%)	7 (38.9%)	0.008
ipsi‐hemi	7 (11.5%)	3 (7.0%)	4 (22.2%)	
Generalized/multifocal	13 (21.3%)	6 (13.9%)	7 (38.9%)	
MRI				
disco‐PQ	47 (77.1%)	36 (83.7%)	11 (61.1%)	0.028
ipsi‐hemi	11 (18.0%)	7 (16.3%)	4 (22.2%)	
both‐hemi	3 (4.9%)	0 (0.0%)	3 (16.7%)	
FDG‐PET				
disco‐PQ	34 (55.7%)	29 (67.4%)	5 (27.8%)	0.005
ipsi‐hemi	22 (36.1%)	13 (30.3%)	9 (50.0%)	
both‐hemi	5 (8.2%)	1 (2.3%)	4 (22.2%)	
SEP and MEP				
Performed	20 (32.7%)	11 (25.6%)	9 (50.0%)	0.064
None	41 (66.3%)	32 (74.4%)	9 (50.0%)	
Pathology				
MCD	42 (68.9%)	31 (72.1%)	11 (61.1%)	0.388
Encephalomalacia	9 (14.8%)	7 (16.3%)	2 (11.1%)	
Encephalitis	3 (4.9%)	1 (2.3%)	2 (11.1%)	
S‐W	1 (1.6%)	1 (2.3%)	0 (0.0%)	
None	6 (9.8%)	3 (7.0%)	3 (16.7%)	
APOS				
Yes	19 (31.1%)	9 (20.9%)	10 (55.6%)	0.008
No	42 (68.9%)	32 (79.1%)	8 (44.4%)	

Abbreviations: both‐hemi, abnormalities in both hemispheres; disco‐PQ, abnormalities confined in disconnected posterior region; ipsi‐hemi, abnormalities beyond disconnected posterior region but in ipsilateral hemisphere.

^a^ Median (range); *n* (%).

^b^ Wilcoxon rank‐sum test; Pearson's Chi‐squared test; Fisher's exact test.

### 3.2 Surgery and pathology

Among the 61 pediatric patients, 32 underwent left‐sided surgery, and 29 underwent right‐side procedures. Interoperative SEP/MEPs monitoring was performed in 20 patients. However, in last 3 years, the increased use of three‐dimensional reconstruction software (Sinovision software, Beijing Sinovision Medical Technology Co., LTD.) has reduced the need of SEP/MEP monitoring during PQD surgery, except when the lesion involved specific areas such as the posterior or anterior central sulcus cortex.

All patients underwent total PQD surgery, with the majority (83.6%) having resection of the posterior regions of the insular cortex, typically the second long insular gyrus, adjacent to the disconnection site resected. In one case, a small portion of the lower central cortex was also resected. Two patients had invasive intracranial EEG electrodes for lesion localization: one underwent subdural electrode implantation, and the other had SEEG. The latter patient continued to experience seizures despite the resection of the parietal lobe lesion, necessitating a total PQD upon reassessment. Another patient, who received VNS following total PQD, also continued to experience seizures postoperatively. MRI scans performed 3 months postoperatively confirmed complete and thorough disconnection of the posterior quadrant in all patients.

Pathologically, 42 patients (68.9%) were diagnosed with MCD, representing more than half of all pathological diagnoses. Six patients did not undergo pathological evaluation due to early‐stage operational factors at our epilepsy center. Other pathological findings included nine cases of encephalomalacia, three cases of encephalitis, and one case of Sturge–Weber syndrome (Table 1). Detailed classification of MCD was provided in Table S1.

### 3.3 Complications

Five patients experienced temporary limb weakness on one side postoperatively; however, their muscle strength returned to normal within 2–4 weeks. There were no cases of permanent postoperative complications, such as hydrocephalus. Unfortunately, during a follow‐up at 3 years post‐surgery, one patient had passed away at home during a seizure status. The specific cause of death was not documented.

### 3.4 Seizure outcome

**FIGURE 1:**
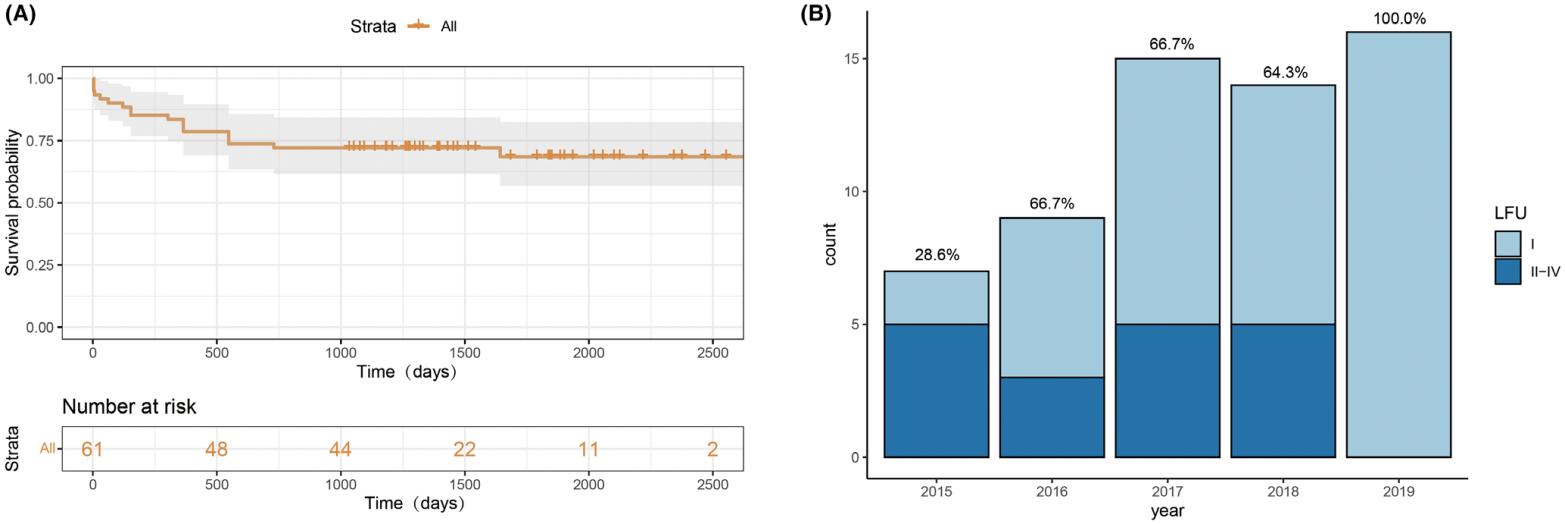
Seizure outcomes: (A) Survival analysis illustrating the chances of post‐surgical seizure freedom; (B) Seizure freedom rate at the last follow‐up by year.

As shown in Figure 1A, the Kaplan–Meier curve reveals that 70.5% of patients were free from seizures after more than 3 years of follow‐up, achieving Engel Class I status. The seizure‐free rate showed a general upward trend over the years, with the highest rates closer to the present time. Notably, in 2019, all patients remained seizure‐free post‐surgery (Figure 1B).

**FIGURE 2:**
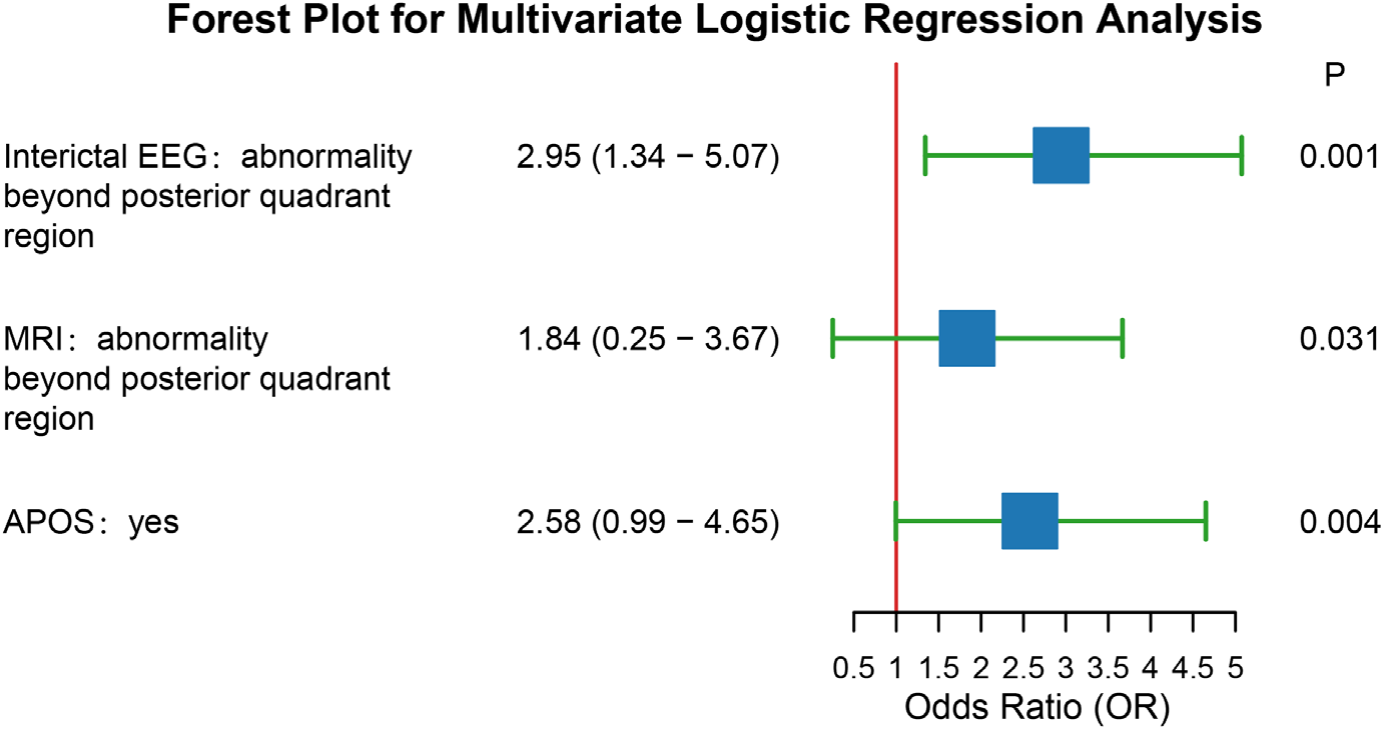
Multivariate logistic regression analysis: Abnormality of interictal EEG beyond posterior quadrant region (OR = 2.95, 95%CI:1.34–5.07, *p* = 0.001), and APOS (OR = 2.58, 95%CI:0.99–4.65, *p* = 0.004) were identified as predictors for PQD surgery treatment.

In the univariate correlation analysis, interictal EEG abnormalities, MRI findings, FDG‐PET results, and APOS occurrence were significantly associated with prognosis (*p* < 0.05). After categorizing MRI and FDG‐PET findings into two groups—those with abnormalities confined to the disconnection area and those involving areas outside the disconnection zone—the four variables with significant differences were subjected to multivariate logistic regression analysis. Following the stepwise regression method, FDG‐PET, which was not significant was removed from the multivariate analysis. Interictal EEG abnormalities, MRI, and APOS were significant (*p* < 0.05), but confidence interval for MRI crossed 1. Therefore, interictal EEG abnormalities and APOS were identified as predictors for PQD surgery treatment (Figure 2).

**FIGURE 3:**
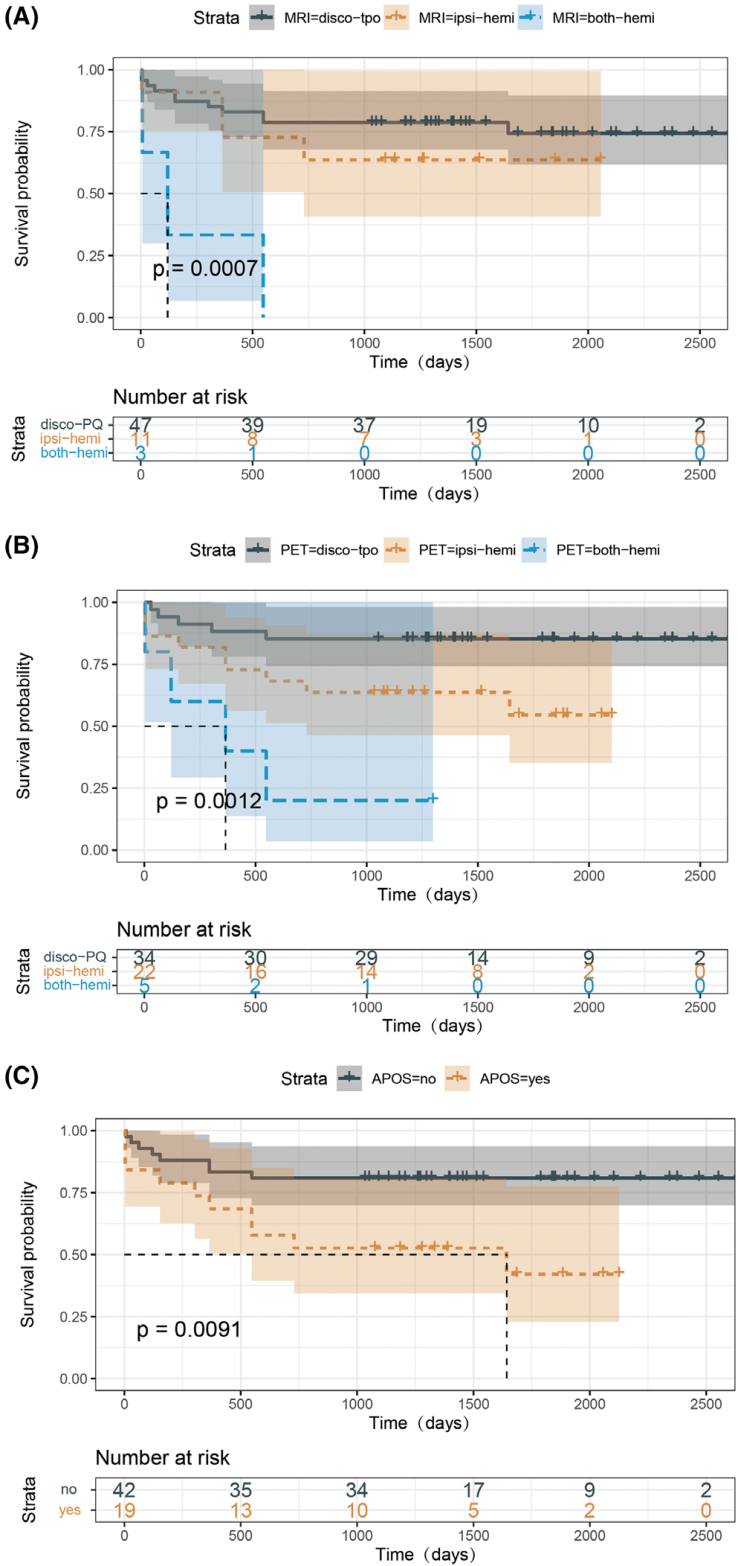
The log‐rank test demonstrated significant differences in (A) MRI findings, (B) FDG‐PET findings, and (C) APOS rate (*p* < 0.01). both‐hemi, abnormalities in both hemispheres; disco‐PQ, abnormalities confined in the disconnected posterior region; ipsi‐hemi, abnormalities beyond the disconnected posterior region but in the ipsilateral hemisphere.

Nineteen patients experienced APOS after surgery. At the last follow‐up, it was found that seven with typical seizures, similar to their habitual presurgical seizure type, continued to have seizures. In contrast, I only 3/12 patients with atypical APOS (non‐habitual seizures) had seizures at the last follow‐up. Most of these patients exhibited contralateral facial or limb convulsions, likely due to stimulation of the surrounding cortex or cerebral tissue edema caused by the surgery. Supportive treatment led to improvement in these cases. The log‐rank test demonstrated significant differences in MRI findings, FDG‐PET results, and APOS occurrence (*p* < 0.01; Figure 3A–C).

### 3.5 Developmental assessment

Seizure frequency (*p* < 0.001), interictal EEG (*p* < 0.001), seizure duration (*p* < 0.05), spasms and FDG‐PET results (*p* < 0.05) were closely related to preoperative developmental levels. Specifically, a higher seizure frequency, longer disease duration, broader interictal EEG abnormality range, the presence of spasms and ipsilateral or contralateral FDG‐PET abnormalities were associated with poorer preoperative developmental levels (Table 2).

**TABLE 2:** Correlation between presurgical GMSD‐C and patient characteristics.

	Presurgical development (*n* = 43)	
	Test statistics	*p*‐value
Surgery age, years^b^	−0.1712	0.2723
Onset age, years^b^	0.1579	0.3119
Duration, years^b^	−0.3257	0.0331*
Semiology^a^		1
Spasms^a^		0.047*
Frequency^a^		< 0.001***
Etiology^a^		0.926
Side^a^		1
EEG onset^a^		1
Interictal EEG^a^		< 0.001***
MRI^a^		1
FDG‐PET^a^		0.021*
SEP and MEP^a^		0.946
LFU^a^		0.91

*Note*: Test statistics: ^a^Fisher's exact test, ^b^Spearman correlation, rho.

Abbreviation: LFU, last follow‐up.

* <0.05.

*** <0.001.

**FIGURE 4:**
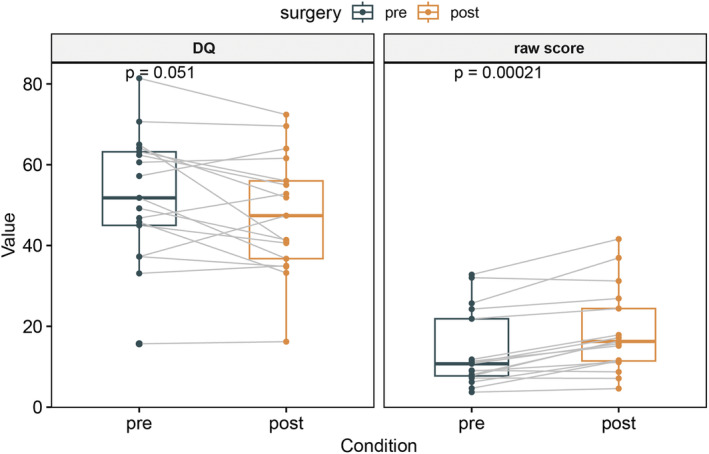
Paired *T* test comparing the pre‐ and postoperative GMDS‐C values: Developmental quotients (DQ; left) and raw scores (right).

Seventeen patients underwent both pre‐ and post‐surgical developmental tests. Postoperatively, there was a significant improvement in raw developmental scores compared to the preoperative scores (*p* < 0.001). However, there were no significant differences in DQ between the preoperative and postoperative values (*p* > 0.05; Figure 4). Similar results were observed in the five domains of GMDS‐C using paired *T* test, excluding DQ in performance domain (Table S2).

## 4 DISCUSSION

DRE due to multilobar lesions is a common occurrence in pediatric patients, particularly within the posterior quadrant region. This study focused on patients who underwent total PQD, with a total case number exceeding 60. All cases had a surgery timeline of less than 5 years, reducing errors related to annual variations. The largest prior study tried to categorize surgical procedures into four types. However, this categorization did not include all types of PQD.^15^ In another study with a relatively large sample of 62 pediatric patients, the procedures only included patients with epilepsy lesions located within the posterior quadrant region.^16^ Most PQD studies include a variety of posterior quadrant disconnection or resection procedures, leading to significant bias when analyzing relevant factors in seizure and developmental outcomes. This study sought to minimize errors by selecting patients who underwent total PQD from 110 patients with posterior quadrant epileptogenic lesions treated by surgery at our center during the same period.

### 4.1 Seizure outcome analysis and prognostic factors

The long‐term seizure‐free rate in this study was 70.5%, which aligns with findings from previous literature on PQD. Rizzi summarized seven previous PQD studies and reported postoperative seizure‐free rates ranging from 25% to 90%.^15^ However, these studies typically had smaller sample sizes. In a review by Markosian et al., nine studies were analyzed, and the postoperative seizure‐free rate for total PQD was reported as 74.6%. One key difference between our data and that of Markosian et al. is that our study exclusively included children, whereas theirs included both children and adults, with their ages ranging from 0.2 to 60.1 years.^6^ Children often present with more complex localization of epileptic foci in both EEG and MRI due to the delayed completion of central nervous system myelination, making the visualization of epileptogenic lesions on MRI more ambiguous.^17^, ^18^ Many MCD patients may have lesions that are located in the posterior quadrant region on MRI, but the underlying pathology might be hemispheric. Among the 18 patients with poor outcomes, 7 had lesions beyond the PQD area, including ipsilateral or both hemisphere involvement. They underwent total PQD because the electroclinical information suggested abnormalities confined in the posterior quadrant region. Three patients with bihemispheric lesions achieved bad seizure outcome at the last follow‐up。Of the 11 patients who had extra‐PQD abnormalities in the ipsilateral hemisphere, 7 (63.6%) were seizure‐free. Among the 7 patients who were seizure‐free, although they had structural abnormalities confined to one hemisphere, their limb functions were not impaired, and their interictal EEG abnormalities were localized to the TPO region. During the preoperative evaluation, we considered a staged surgical approach. If the first surgery failed, a second surgery, potentially involving limb function compromise, would be considered for a curative outcome. MRI abnormalities outside the surgical regions are not always epileptogenic foci and a comprehensive consideration of all examinations, including EEG and FDG‐PET, is necessary to determine the final surgical plan. Therefore, refusing curative surgery should not be solely based on the presence of structural lesions beyond the PQD. Despite the possibility of seizure recurrence, a prolonged seizure‐free period was favorable for child development. Excluding 2015, where the postoperative seizure‐free rate was only 28.6% (7 patients), the rate remained above 60% in the following years and notably reached 100% in 2019. The year‐by‐year increase in the seizure‐free rate is a common phenomenon observed in previous literature, mainly attributed to a learning curve for the surgical technique, reducing the incidence of residual lesions due to incomplete disconnection.^6^ Additionally, patients with shorter follow‐up times tend to have a higher seizure‐free rate. Enhanced preoperative assessment, which involves selecting suitable surgical candidates, also contributes to improve the postoperative seizure freedom rate.

The preoperative assessment of pediatric PQD patients follows the same anatomical–electroclinical principles as in adults, but it is often more complex. In this study, all patients underwent FDG‐PET scans. In previous literature, the rate of metabolic functional checkups, including FDG‐PET /SPECT, was only 28.5%, as reported by Markosian C,'s in their review of 137 cases from nine studies published in the recent past 20 years.^10^ FDG‐PET is significantly advantageous in detecting the seizure focus, especially in cases with potential structural anomalies on MRI and widespread multifocal EEG abnormalities.^19^ In this study, only 57% of the patients had FDG‐PET abnormalities confined to the disconnection area, consistent with hypometabolism that extends beyond the seizure‐onset zone described in previous literature.^19^ While FDG‐PET results showed a correlation with outcomes in the univariate analysis, their higher sensitivity in detecting lesions led to the identification of more abnormalities. However, when included in the multivariate analysis along with other variables, the predictive power of PET was diluted due to the presence of these additional abnormalities. Therefore, PET was not considered a significant predictive factor in the multivariate analysis.

In children, interictal EEG abnormalities tend to be more widespread than in adults. In our cohort, 32.8% of the patients had EEG abnormalities outside the disconnection areas. Our results demonstrate that the location of abnormal interictal EEG discharges is a predictive factor for postoperative seizures. Specifically, patients with interictal discharges in the posterior quadrant region have better outcomes. In studies involving both children and adults, the consistency between interictal and ictal EEG features is a predictive factor for being seizure‐free.^10^ However, because childhood spasm seizures are relatively common even in cases of focal seizures, the location of the ictal EEG in children is not related to outcomes, consistent with our previous research findings.^20^ The presence of spasms presence often leads to bilateral symmetrical typical epileptic spasm and/or diffusive and multifocal discharges, making it difficult of identifying the location epileptogenic foci.^20^ However, this characteristic of epileptic spasms should not be a reason to refuse surgical treatment.^20^

APOS, whether in adult or pediatric studies, is considered a common prognostic factor.^14^, ^21^, ^22^ Following PQD, a relatively common form of postoperative seizures is characterized by twitching at the corner of the mouth on the contralateral side of the surgery, sometimes accompanied by limb trembling. This occurs due to the proximity of the surgical disconnection site to the motor cortex and edema of the surrounding tissue. This condition is usually transient and disappears within 7–8 days after surgery due to the resolution of postoperative brain tissue edema.

### 4.2 Complications

In PQD, the anterior boundary is the posterior central sulcus, and some children may experience short‐term contralateral limb weakness symptoms after surgery. This weakness typically resolves, with strength returning to normal within 2–4 weeks, which is consistent with previous reports.^6^, ^12^, ^23^ This phenomenon is likely related to surgical experience. In most cases, it is due to suboptimal direction control during the disconnection process, leading to mild damage or temporary edema of the corticospinal tracts. Previous literature has emphasized the importance of central area mapping for the success of surgery.^12^ However, by using image processing software for preoperative 3D reconstruction, precise control of the disconnection direction during surgery, and careful protection of the arcuate bundle when disconnecting from the postcentral sulcus to the white matter, surgery can be accomplished without causing any postoperative limb weakness. Monitoring of SEPs and MEPs during surgery is indicated when the lesion extends into the lower part of the central operculum or the posterior part of the insular lobe.

Most children are unable to complete visual field testing. In some children with posterior quadrant‐associated lesions, visual field defects may have existed preoperatively.^10^ Our study did not discuss visual field issues.

For PQD in the dominant hemisphere, Rizzi chose to preserve the Wernicke area to prevent postoperative language deficits.^15^ In patients with perinatal stroke, diminished language function in the dominant hemisphere often leads to significant compensation by other brain regions.^24^ Additionally, MCD occurs during pregnancy, and functional compensation may exist when MCD influences the dominant hemisphere. In our study, a consistent surgical approach was adopted for all children with lesions involving the dominant hemisphere, regardless of the underlying cause, without considering the preservation of the Wernick cortex. None of the patients showed regression in language comprehension or expression after surgery.

### 4.3 Development analysis

There is currently no literature reporting the impact of PQD surgery on development. In our study, the preoperative characteristics were found to be correlated with the presurgical developmental level. Patients with higher seizure frequencies, longer disease durations, broader interictal EEG abnormality ranges, the presence of spasms, and larger FDG‐PET abnormality ranges had lower developmental levels. This is consistent with the results of some retrospective analyses in studies that are not specific to PQD.^14^ Among these influencing factors, only the disease duration is modifiable in that it can be shortened through surgical treatment. This further proves the advantage of early epilepsy surgery and emphasizes the importance of comprehensive presurgical evaluation as early as possible.

By comparing developmental levels before and after surgery, we found that the raw scores on the five domains (locomotor, personal social, language, eye‐hand coordination, performance) of GMDS‐C at 3 months postoperatively were significantly higher than those preoperatively, despite a slight decrease in DQ. This aligns with some previous research conclusions.^25^, ^26^ However, given the short follow‐up period of 3 months, these findings should be interpreted with caution. Although 3 of 17 patients in this group still had seizures after surgery, which were classified by the ENGEL classification as II‐III, the reduced frequency of seizures appeared to provide some developmental benefits. Further long‐term studies are necessary to confirm these preliminary observations.

## 5 CONCLUSIONS

For patients with structural abnormalities involving the entire posterior quadrant area, careful preoperative assessment and timely total PQD surgery can effectively terminate or reduce the frequency of seizures without deterioration of neurological development. The occurrence of APOS and interictal EEG abnormalities outside posterior quadrant regions are predictive factors for seizure prognosis. However, these predictive factors should not be considered contraindications for surgery.

## 6 LIMITATIONS

This study has several limitations, including a small sample size, single‐center design, and short follow‐up period. Additionally, not all patients had complete developmental assessment data due to different reasons, such as long distances to our hospital, economic difficulties, and other factors. The study would be more comprehensive if these data were complete.

## AUTHOR CONTRIBUTIONS

LC designed this study. HY analyzed the data and drafted and revised the manuscript. CL, YS and YW helped to select the patients. QL collected the data. XL, YJ and WY revised the manuscript. TJ and SW helped to interpret the EEG data. All the authors contributed to the article and approved the submitted version.

## CONFLICT OF INTEREST STATEMENT

None of the authors have any conflicts of interest to disclose. We confirm that we have read the Journal's position on issues involved in ethical publication and affirm that this report is consistent with those guidelines.

## Supporting information


Table S1.
Table S2.

## Data Availability

The raw data supporting the conclusions of this article can be obtained from the authors upon reasonable request.
