# Supplementary material for: Total posterior quadrant disconnection for drug‐resistant epilepsy in children
Source: Epilepsia Open. 2024 Sep 19;9(6):2198–208. doi: 10.1002/epi4.13044 (PMC11633695; doi:10.1002/epi4.13044)
Supplement: Supplementary file 1 — Table S1. Table S2. [file EPI4-9-2198-s001.docx]

**Table1 Pathological substrates classified as malformation of cortical development (MCD) according to ILAE classification**

| **MCD substrates** | **Number(total=42)** |
| --- | --- |
| Ia | 6 |
| Ib | 7 |
| IIa | 9 |
| IIb | 4 |
| Other MCDs | 16 |

**Table 2 The changing in 5 domains of GMSD-C between pre- and 3-months-post- surgery using paired T-test**

| **Paired T test** | **Locomotor** | **Personal social** | **Language** | **Eye-hand coordination** | **Performance** |
| --- | --- | --- | --- | --- | --- |
| **P（raw score）** | <0.001 | <0.001 | <0.001 | 0.001 | 0.022 |
| **P（DQ）** | 0.536 | 0.057 | 0.241 | 0.117 | 0.034 |

DQ：developmental quotients
